# Individual variation in spawning migration timing in a salmonid fish—Exploring roles of environmental and social cues

**DOI:** 10.1002/ece3.10101

**Published:** 2023-05-17

**Authors:** Michio Fukushima, Peter S. Rand

**Affiliations:** ^1^ Biodiversity Division National Institute for Environmental Studies Ibaraki Japan; ^2^ Prince William Sound Science Center Cordova Alaska USA

**Keywords:** behavioral repeatability, coordinated movement, environmental cue, migratory connectivity, Sakhalin taimen, social interaction

## Abstract

Describing and explaining patterns of individual animal behaviors in situ, and their repeatability over the annual cycle, is an emerging field in ecology owing largely to advances in tagging technology. We describe individual movements of adult Sakhalin taimen *Parahucho perryi*, an endangered salmonid fish, in the headwaters of a river in northern Japan during the spring spawning season over 2 years. Migration timing, separated into stages prior to, during, and following the spawning period, was found to be more consistent and repeatable for females than males. We hypothesized that the observed coordinated movement within seasons, and repeatability in migration timing across seasons, could result from (1) individual‐specific responsiveness resulting from endogenous, biological traits that are mediated by environmental factors, or (2) social interactions among comigrating individuals. We found that water temperature and water level experienced by fish near the river mouth approximately a week before arrival at the spawning ground explained variability in run timing between years for females but not males. We found no evidence of conspecific attraction or repulsion resulting from social interactions among the spawners and post‐spawners. We conclude that individual‐specific responsiveness to environmental cues was the likely mechanism underpinning the observed migration timing and movement patterns.

## Graphical abstract

Individual‐specific responsiveness to environmental cues was the likely mechanism underpinning the coordinated movement and across‐year repeatability in migration timing in the endangered salmonid, Sakhalin taimen. The species possess strong fidelity to time of reproductive activities to maintain their migratory connectivity.

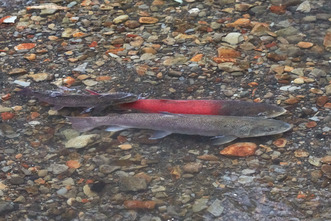

## 1 INTRODUCTION

Migratory species often exhibit coordinated movements underpinned by individual‐specific timing of migration, whereby individuals arrive consistently early or late, relative to other individuals in a population, at specific habitats or life‐history stages (Bell et al., 2009; Biro & Adriaenssens, 2013; Tamario et al., 2019). Timing of migration has proven to be crucial in defining fitness consequences related to reproductive success and offspring survival (Brodersen et al., 2012; Tibblin et al., 2016). Intra‐individual consistency in animal movements, social coordination, and repeatability in migration timing have received increased scientific attention (Herbert‐Read, 2016; Westley et al., 2018). Advanced tracking technologies involving biologging and telemetry have provided empirical evidence for these behavioral traits in many migratory species, particularly birds (Gilsenan et al., 2020; Kentie et al., 2017) and fishes (Brodersen et al., 2012; Forsythe et al., 2012; van Wijk et al., 2016).

Migration timing has been shown to be partially determined by an endogenous, circannual rhythm that synchronizes behaviors with photoperiod in birds and fishes (Eriksson & Lundqvist, 1982; Pulido et al., 2001; Styrsky et al., 2004). This rhythm arises from genetic evolution in animals, and it is therefore important to recognize that migratory behaviors are heritable and best understood in the context of evolutionary adaptation (Brönmark et al., 2013; Quinn et al., 2000; Thompson et al., 2019). Although these rhythms are driven by endogenous hormone cycles cued by photoperiod, migration timing in individuals can be affected by environmental factors (Franklin et al., 2022; Harrison et al., 2017). Because of consistent individual differences in animal behavior (Biro & Adriaenssens, 2013; Nilsson et al., 2014), the behavioral traits of individuals responding to environmental controls with pronounced seasonality (e.g. annual temperature dynamics in temperate ecosystems) tend to be repeatable across years (Dahl et al., 2004; Gilsenan et al., 2020; Quinn et al., 1997). However, identifying proximate environmental drivers and quantifying their effects on migratory behaviors have been technically difficult, especially in a field setting, because researchers generally can only recognize migration at its end point (e.g. a breeding site), and not at the time and location at which the migration was initiated (Porlier et al., 2012; Winkler et al., 2014). An additional complication is that animals typically migrate when environmental variables are correlated and monotonically increase (or decrease), inevitably leading to spurious correlations between some variables and the day‐of‐year of migration, even though no causal mechanisms exist (Dahl et al., 2004; Sinnatamby et al., 2018).

Alternatively, repeatability in migration timing could be manifested when animals form cohesive groups (e.g. bird flocks and fish schools) of socially interacting individuals with strong, long‐lasting group fidelity (Fraser et al., 2005; Hay & McKinnell, 2002; Klimley & Holloway, 1999). Group migration, or collective navigation, is known to facilitate rapid transfer of beneficial behavioral traits (Gil et al., 2018; Thorsteinsson et al., 2012), help avoid predation, correct for errors made by less experienced members (Berdahl et al., 2016), and improve homing precision to natal locations (Bett & Hinch, 2015). This mechanism, however, may not be entirely independent of the environmental control mechanism described above because at least some group members would still rely on external cues for decision making (Couzin, 2009; Herbert‐Read, 2016; Herbert‐Read et al., 2017). Even though the social aspect of animal migration has been investigated in laboratory and theoretical studies, it remains largely unexplored in field‐based ecological studies because of logistical and methodological constraints (Brönmark et al., 2013; Gil et al., 2018).

**FIGURE 1:**
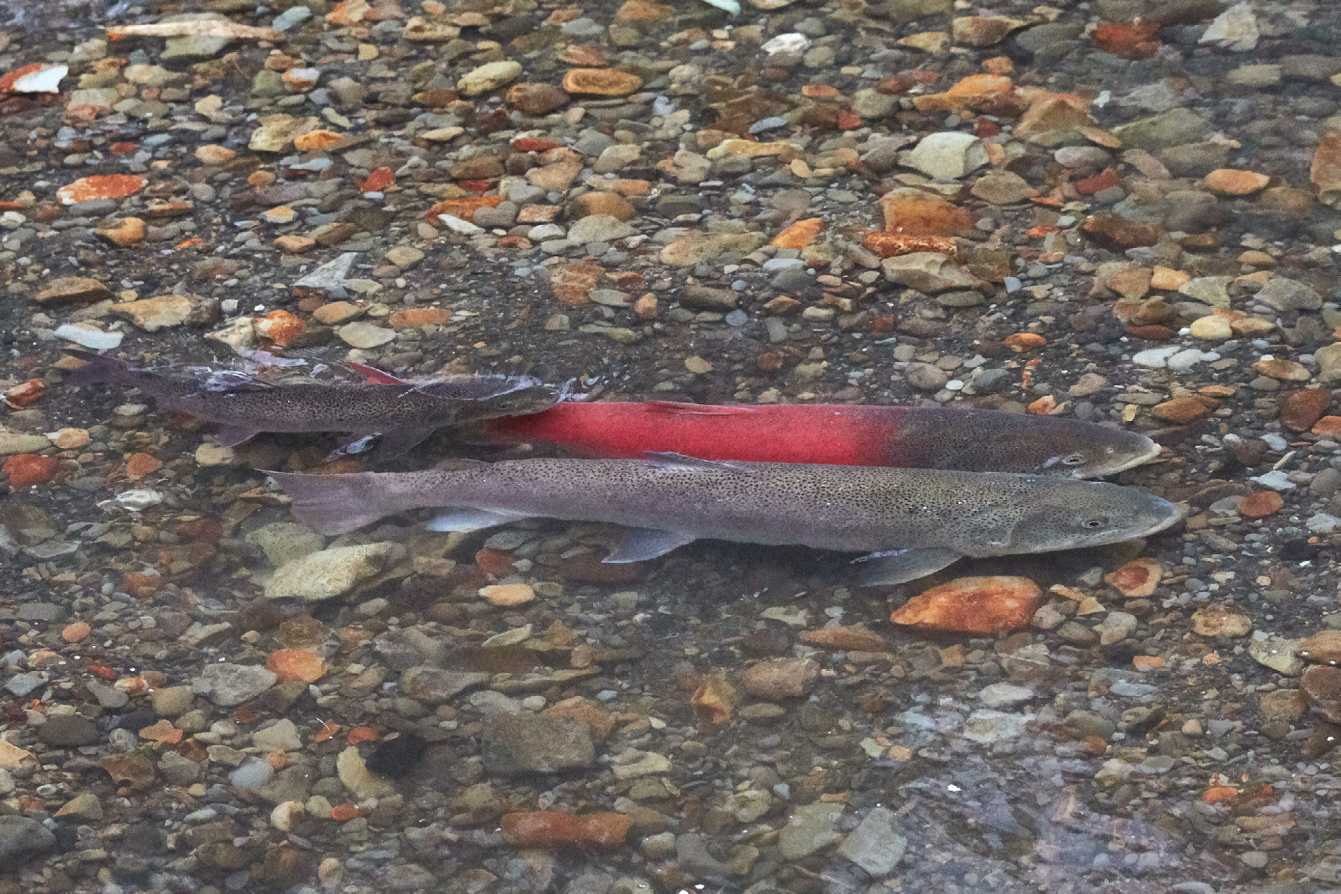
A pair of Sakhalin taimen on the spawning ground, with a subordinate male (left in image). Note the dominant male is in reddish, nuptial color.

Sakhalin taimen *Parahucho perryi* (Figure 1) is a critically endangered, long‐lived (>25 years), iteroparous salmonid inhabiting far eastern Russia and Hokkaido, Japan (Rand, 2006). During the spring spawning season, mature Sakhalin taimen migrate upstream to headwater streams in small groups from their non‐breeding habitats in the estuary (Rand & Fukushima, 2014). A recent tagging study revealed that up to 87% of Sakhalin taimen spawners return to the same tributaries across consecutive years, one of the highest rates of site fidelity ever documented for iteroparous salmonids (Fukushima & Rand, 2021). The group migration and strong site fidelity of the species are consistent with the hypothesis that social interactions influence their movement patterns during the spawning migration. However, the rate of upstream migration of this species is significantly influenced by water temperature and stream discharge (Rand & Fukushima, 2014), suggesting the importance of environmental controls on migration timing as well.

In this study, we investigated the migratory behavior of adult Sakhalin taimen by tracking spawners and post‐spawners in a river system in Hokkaido, Japan, with multiple spawning streams. We first characterized migration timing at specific waypoints and examined the degree to which the migration timing was repeatable among individuals across years. We then explored whether the repeatability in migration timing resulted from individual‐specific responsiveness to seasonality in the environmental controls, or from social interactions among comigrating individuals. We discuss our findings in relation to the concepts of migratory connectivity (Webster et al., 2002) and differential migration (Briedis & Bauer, 2018; Brodersen et al., 2012).

## 2 MATERIALS AND METHODS

### 2.1 Fish sampling and tagging

**FIGURE 2:**
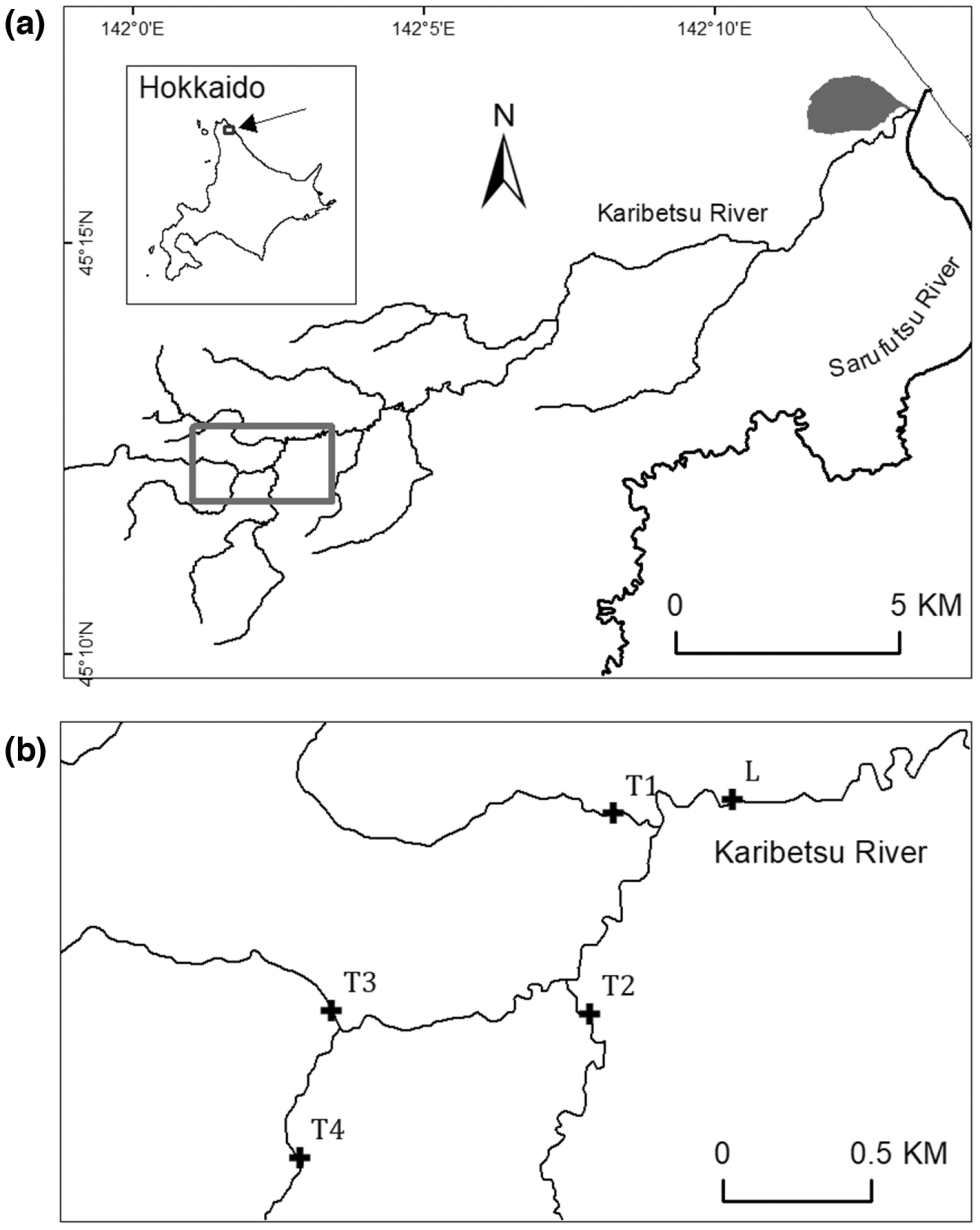
(a) Map of the Karibetsu and Sarufutsu Rivers in Hokkaido, Japan. The gray rectangle denotes the area shown in (b). (b) Locations of fish sampling (L) and passive integrated transponder (PIT) detection systems (L and T1–T4) in the Karibetsu River.

During the spring spawning seasons in 2016, 2017, and 2018, we captured a total of 123 Sakhalin taimen spawners (67 females and 56 males) in the Karibetsu River (watershed area: 83 km^2^), a tributary of the Sarufutsu River in northern Hokkaido, Japan (Figure 2). The fish were sampled daily with a cast net as they ascended a fish ladder adjacent to a weir (approx. 1 m high × 12 m wide) located approximately 22 river kilometers (rkm) from the ocean (hereafter, referred to as Site L). Note that this site marks an approximate downstream boundary of the Sakhalin taimen spawning ground in the Karibetsu River. Each fish captured was anesthetized with eugenol solution (approx. 30 ppm; FA100, Pharma Animal Health Co., Ltd.), sexed, measured for fork length (FL), marked with a 23‐mm passive integrated transponder (PIT) tag, and released above the weir. The number of tagged fish represented 29%–37% of the annual run of the species in this river according to the estimates obtained by using underwater imaging sonar (Rand & Fukushima, 2014). Fish were collected under Hokkaido Government permits No. 168 (2016), 201 (2017), and 191 (2018). The fish sampling protocol used in this study was approved by the Animal Care Committee of the National Institute for Environmental Studies.

In 2018 and 2019, antenna arrays (Oregon RFID) were installed to detect PIT‐tagged fish at Site L and four tributary sites (T1–T4) located 0.79–3.53 rkm upstream from Site L (Figure 2). One pass‐through antenna was built across the stream at each tributary site, whereas one pass‐through antenna and an additional pass‐over antenna were built side‐by‐side at Site L at the upstream exit of the fish ladder and across the spillway, respectively, to detect both ascending spawners and descending post‐spawners. Arrival timings at Site L of the 90 fish captured there in 2018 are represented by the time of their capture for tagging. These fish were handled only briefly before release upstream and were captured roughly in the order they arrived at this site.

The swimming speed of Sakhalin taimen during upstream and downstream migrations was estimated by dividing the distances traversed between Site L and tributary sites by differences in the respective detection times in 2019, when there was no fish sampling (i.e. no effects of tagging on fish migration). The upstream migration speed, which was log‐normally distributed, was then used to calculate the mean and 95% confidence interval of the total travel time of the spawners from a presumed starting point at the river mouth to a point at 24.2 rkm, the average distance to the tributary sites. Further details on the PIT tagging and detection are described by Fukushima and Rand (2021).

Water temperature and water level were monitored at Site L throughout the 2018 and 2019 spawning seasons at 10‐min intervals using temperature and pressure loggers (UA‐001‐64 and U20‐001‐01, respectively; Onset Computer Corporation). Two pressure loggers—one underwater and the other on the riverbank—were deployed to correct for changes in atmospheric pressure.

### 2.2 Repeatability in migration timing

We calculated Spearman's rank correlation between migration timings in 2018 and 2019 for individuals that returned consecutively to the Karibetsu River. The calculation was made on both seasonal (date and time) and diel (time only) scales, separately for each sex at each of the following migration stages: arrival at Site L from the estuary (migration stage [MS]1), arrival at tributary sites from Site L (MS2), departure from tributary sites for Site L (MS3), and departure from Site L for the estuary (MS4). Seasonal‐scale correlation was also calculated between MS1 and MS4 migration timings of the same individuals in 2019 (i.e. before and after spawning within a season). Circular statistical analyses were performed to test for the uniformity of the diel migration timing using the Rayleigh test, and for differences in the timing between sexes and between upstream and downstream migrations using the Watson–Wheeler test.

### 2.3 Modeling migration timing with biological and behavioral variables

Seasonal migration timing was modeled with linear mixed‐effects models (LMMs) separately for each migration stage. The PIT code was used as a random effect to account for between‐year correlation of the same individuals. Sex, FL, whether migration occurred during daytime (0420–1837 on 1 May, the approximate midpoint of the run, in Sarufutsu Village) or night‐time (DN), and the first (First) and total number of tributaries (Tributary) each fish entered for spawning each year were included as candidate fixed effects. FL was standardized to values in spring 2018 when most fish (90) were measured. Length of individuals measured only before 2018 was estimated based on their original measurements and an estimated annual growth rate of 24.7 mm year^−1^ (Fukushima & Rand, 2021). Sex and FL were constant for each fish across years and MS; First and Tributary could be different across years but constant across MS; and DN could be different across years and MS. To account for possible sex‐related influences, we created a full model including interaction terms between Sex and the other fixed effects. To facilitate comparisons among models, the original response variable of timing in date and time was standardized to a mean of zero and standard deviation of 1 for each year and MS. Water temperature and water level variables were not included in this modeling to avoid spurious correlations described earlier.

### 2.4 Testing the environmental control hypothesis

To determine whether Sakhalin taimen exhibited individual‐specific responsiveness to environmental signals, and when the signals became critical, we used a sliding window approach. We defined a critical time window as a period when an environmental cue perceived by the same individuals between years yielded the highest correlation, based on the premise that the cue to initiate movement should be similar within individuals across years if responsiveness to the signal is idiosyncratic. As such, we calculated a series of Pearson correlations between 2018 and 2019 for each of the two variables (water temperature and water level), averaged over time windows relative to each spawner's arrival timing at the first tributary each year (i.e. MS2). The window size was varied from 8 to 240 h at 4‐h increments in both time lag (time to the window's start) and duration (window width). The observed maximum correlation coefficient (*r*_obs_) and the corresponding critical time window were recorded for each sex and variable. The significance of *r*_obs_ was determined with a randomization test, in which Pearson correlations were calculated between randomly selected individuals from the 2 years by varying the window size to find a critical window with a simulated maximum correlation (*r*_sim_). This process was iterated 999 times, and the proportion of *r*_sim_ as large as or larger than *r*_obs_ was considered a measure of significance by applying a one‐sided test (van de Pol et al., 2016).

### 2.5 Testing the social interaction hypothesis

We evaluated whether Sakhalin taimen employed social navigation strategies that could lead to coordinated movements and repeatable migration timing by asking two questions: (1) did comigrating groups of pre‐spawners ascending through Site L (MS1) tend to enter the same spawning tributaries, diverge into different tributaries, or enter tributaries at random, and (2) did comigrating groups of post‐spawners descending through Site L (MS4) tend to consist of individuals that spawned in and departed from the same tributaries, different tributaries, or any tributary at random? If two or more tagged individuals passed Site L over an elapsed time of <1 h, the individuals were considered ‘comigrants’ according to a time‐series analysis of fish passage in an earlier study (Rand & Fukushima, 2014). Given the disparity in migratory behavior between sexes (see Section 3), we considered sexes separately in this analysis.

To answer the questions above, we counted the observed total number of comigrating pairs during MS1 or MS4 that subsequently entered, or previously departed from, the same tributaries (*n*_obs_) based on the 2019 dataset. Individual migrants were then randomly assigned to a spawning tributary while preserving their actual MS1 (and MS4) migration timings and the total numbers of migrants accommodated by each tributary. Comigrating pairs that were assigned the same tributaries during each randomization trial were counted (*n*_sim_). This process was iterated 9999 times to generate a distribution of *n*_sim_ to compare with *n*_obs_. If *n*_obs_ was found to be significantly high relative to the distribution of *n*_sim_, this would provide evidence that individuals exhibit conspecific attraction during the pre‐spawn (i.e. transition from MS1 to MS2) or post‐spawn (i.e. transition from MS3 to MS4) periods. Conversely, if *n*_obs_ was found to be lower than expected by chance, this provided evidence that individuals exhibit conspecific repulsion during the aforementioned migration stages. Thus, the social interaction hypothesis was tested with two‐sided tests.

All statistical analyses were performed using R version 3.4.3 (R Development Core Team, 2017). The R package ‘lme4’ (Bates et al., 2015) was used to perform LMM, and ‘lmerTest’ (Kuznetsova et al., 2020) was used to select the best models via sequential backward elimination of non‐significant effects, and to calculate *p* values based on Satterthwaite's approximation. Circular statistical analyses used the R package ‘circular’ (Agostinelli & Lund, 2017). Statistical significance was defined at *α* = .05 for all analyses.

## 3 RESULTS

### 3.1 Seasonal migration timing of Sakhalin taimen

In 2018, a total of 105 PIT‐tagged Sakhalin taimen spawners returned to the Karibetsu River, including 90 fish tagged that year. In 2019, 76 tagged spawners returned, of which 73 had also returned in 2018 (i.e. consecutive repeat spawners). Upon capture, mean lengths (FL) of female and male spawners were 772 ± 92 mm and 720 ± 139 mm (mean ± SD), respectively. During the period of upstream migration, water temperature increased monotonically, while water level varied more stochastically but decreased substantially by the time spawners arrived at Site L (Figure S1).

### 3.2 Between‐year consistency in migration timing

**FIGURE 3:**
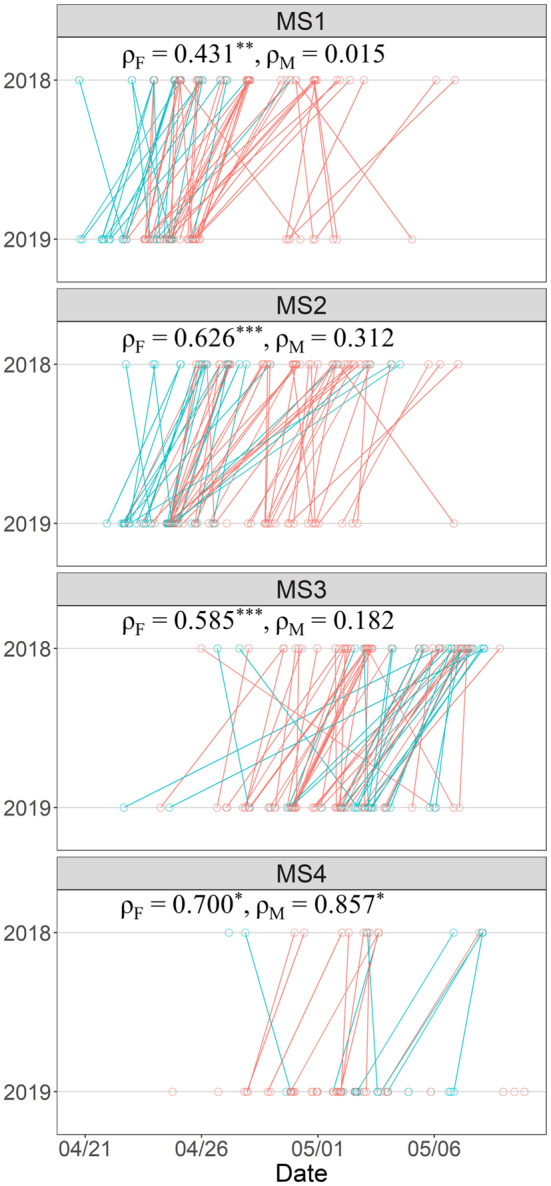
Seasonal migration timing of Sakhalin taimen during four migration stages (MS) in 2018 and 2019. Circles indicate the observed migration timings of females (red) and males (blue) with lines connecting the same individuals across the years. Spearman's rank correlation and its significance (*p < .05, **p < .01, ***p < .001) are shown for females (*ρ*_F_) and males (*ρ*_M_).

The chronological order of individual Sakhalin taimen arriving or departing during each MS was remarkably consistent between 2018 and 2019. Lines connecting the same individuals, especially females, in these 2 years tended to be parallel to one another (Figure 3).

Between‐year correlations of the seasonal migration timings were significantly high for female Sakhalin taimen at all MSs but were significant only at MS4 (*p* = .024) for males. For the females, within‐season correlation between MS1 and MS4 timings was also significant (*ρ* = .628, *p* < .001), but it was extremely low and not significant for the males (*ρ* = .002, *p* = .996). The reason that there are fewer lines for MS4 in Figure 3 is because we terminated PIT detection before all post‐spawners had migrated downstream past Site L in 2018. Only in the females during MS3 was there a significant between‐year correlation on the diel‐scale as well (i.e. female individuals departed from tributaries at similar times of day in both years) (*ρ* = .497, *p* = .001).

**FIGURE 4:**
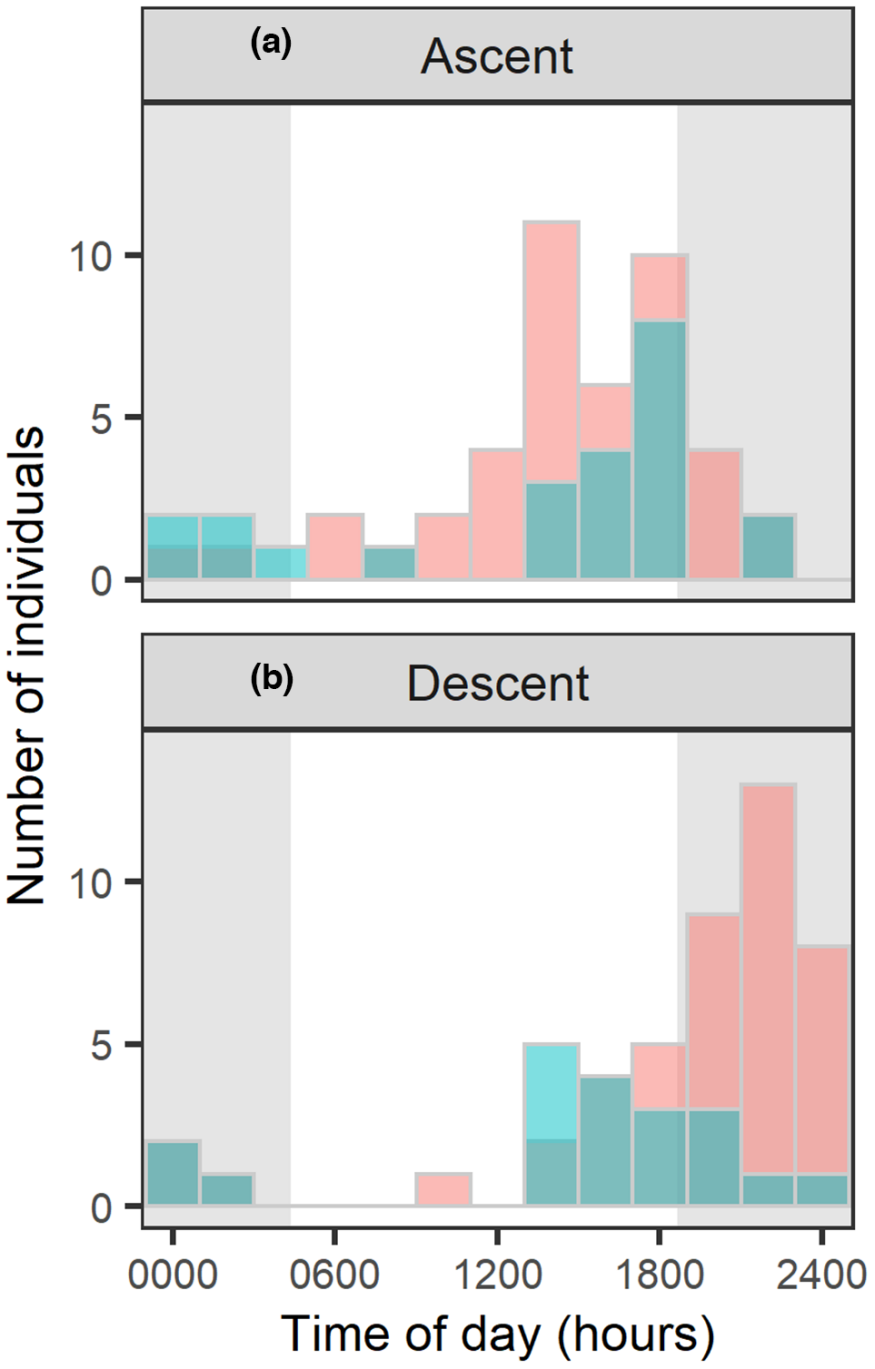
Diel‐scale migration timing of female (red) and male (blue) Sakhalin taimen spawners (a) and post‐spawners (b) in 2019 during ascent at MS1 and descent at MS4, respectively. Gray shading indicates approximate night‐time (1837–0420).

Neither ascent (MS1) nor descent (MS4) by Sakhalin taimen through Site L was uniformly distributed throughout the day (Figure 4, Rayleigh test, *p* < .001 for females, and *p* < .01 for males). Both female and male spawners ascended the river mostly during daytime, peaking before sunset. Descent by post‐spawners peaked early in the afternoon in males, but it peaked during night‐time in females. Consequently, the diel migration timing of female Sakhalin taimen differed significantly between ascent and descent (*p* < .001, Watson–Wheeler test), and the timing of descent differed between the sexes (*p* = .003).

### 3.3 Biological and behavioral effects on migration timing

Sex was a significant fixed effect influencing seasonal migration timing during MS1, with male spawners arriving at Site L several days earlier than females in both years (*p* < .001, Table 1, Figure 3). Males also arrived earlier than females at tributary sites (MS2, *p* < .001). Arrival timing at this stage also depended on which tributaries spawners entered first, with those entering T2 and T4 being the earliest and latest, respectively (*p* = .012, *F*‐test). During MS1, MS2, and MS3, spawners and post‐spawners tended to migrate during night‐time later in the season (*p* = .011, .027, and .006, respectively), which was especially true for females at MS1 (*p* = .010). Individuals that spawned in multiple tributaries tended to depart later during MS3 from the last tributary than those spawning in one tributary (*p* = .031). Sex was the only fixed effect to explain downstream migration timing at MS4, with the female post‐spawners preceding the males (*p* = .009).

**TABLE 1:** Best linear mixed‐effects models to explain the seasonal migration timing of Sakhalin taimen during four migration stages (MS). Sex_m_ and DN_n_ denote Sex = male and DN = night, respectively. First_2_, First_3_, and First_4_ are the effects of entering tributaries T2, T3, and T4 relative to entering T1, respectively.

Stage	Fixed effect	Estimate	SE	df	*t*‐Value	*p*
MS1	Sex_m_	−0.90	0.16	113.02	−5.64	<.001
	DN_n_	0.54	0.21	108.33	2.59	.011
	Sex_m_:DN_n_	−0.89	0.34	109.73	−2.64	.010
MS2	Sex_m_	−0.98	0.16	101.58	−6.29	<.001
	First_2_	−0.13	0.16	161.49	−0.78	.437
	First_3_	−0.08	0.23	158.17	−0.32	.750
	First_4_	0.42	0.20	163.70	2.06	.041
	DN_n_	0.31	0.14	140.64	2.24	.027
MS3	Tributary	0.39	0.18	161.87	2.17	.031
	DN_n_	0.41	0.15	158.78	2.81	.006
MS4	Sex_m_	0.65	0.24	65.31	2.68	.009

### 3.4 Individual‐level environmental control

**FIGURE 5:**
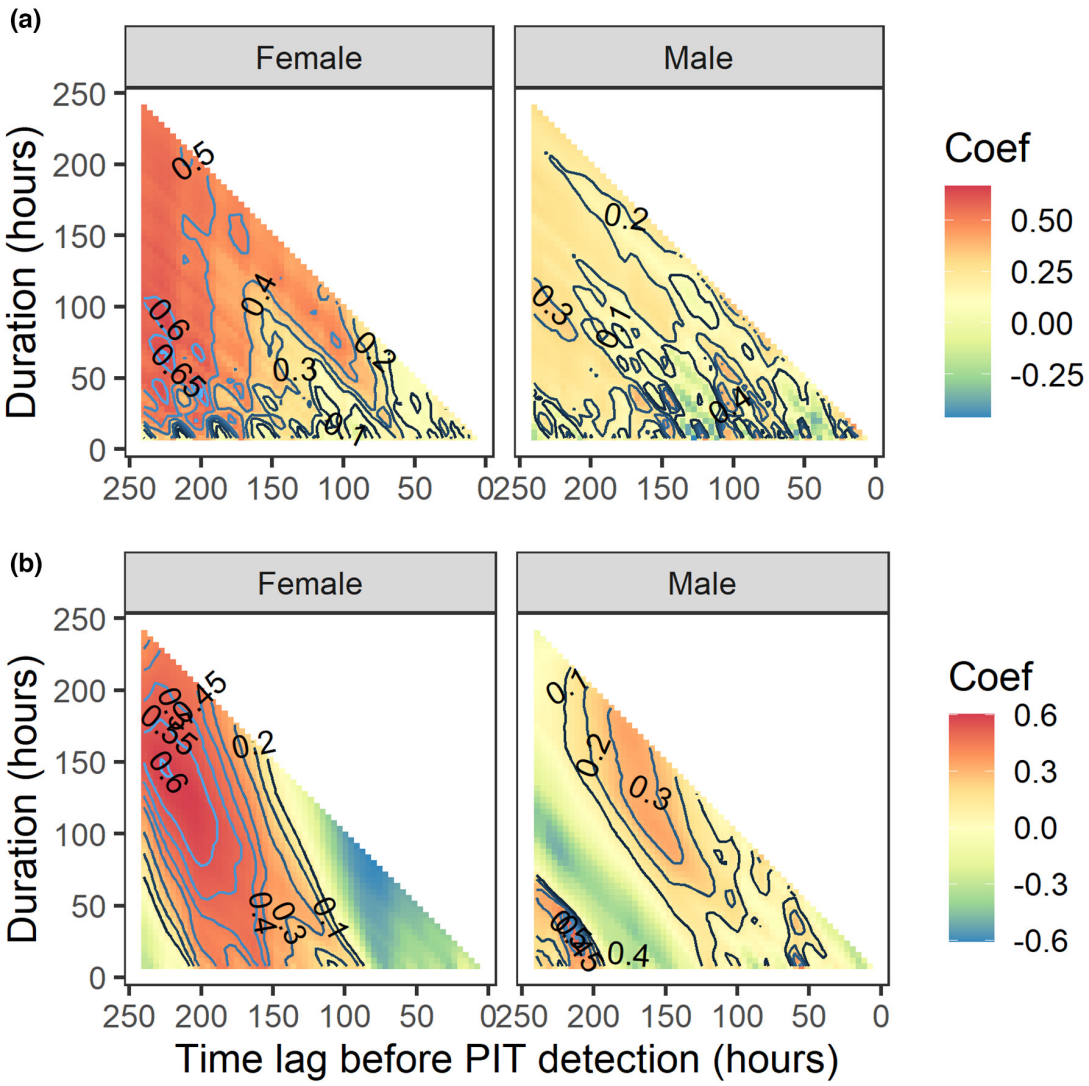
Between‐year correlations of water temperature (a) and water level (b). Time windows represented by time lag and duration are relative to the arrival timings determined by PIT tag detections of the same Sakhalin taimen individuals at tributary sites during MS2 in 2018 and 2019. PIT, passive integrated transponder.

For female Sakhalin taimen, between‐year correlations of water temperature reached a maximum in the critical time window of 160–200 h (6.7–8.3 days) prior to each spawner's arrival at a tributary site (*r* = .665, *p* = .001, Figure 5a). The maximum correlation of water level occurred in the critical time window of 80–224 h (3.3–9.3 days) for the females (*r* = .602, *p* = .014, Figure 5b). For males, however, neither water temperature nor water level produced significant maximum correlations (*r* = .484 and .508, respectively; *p* > .999).

**FIGURE 6:**
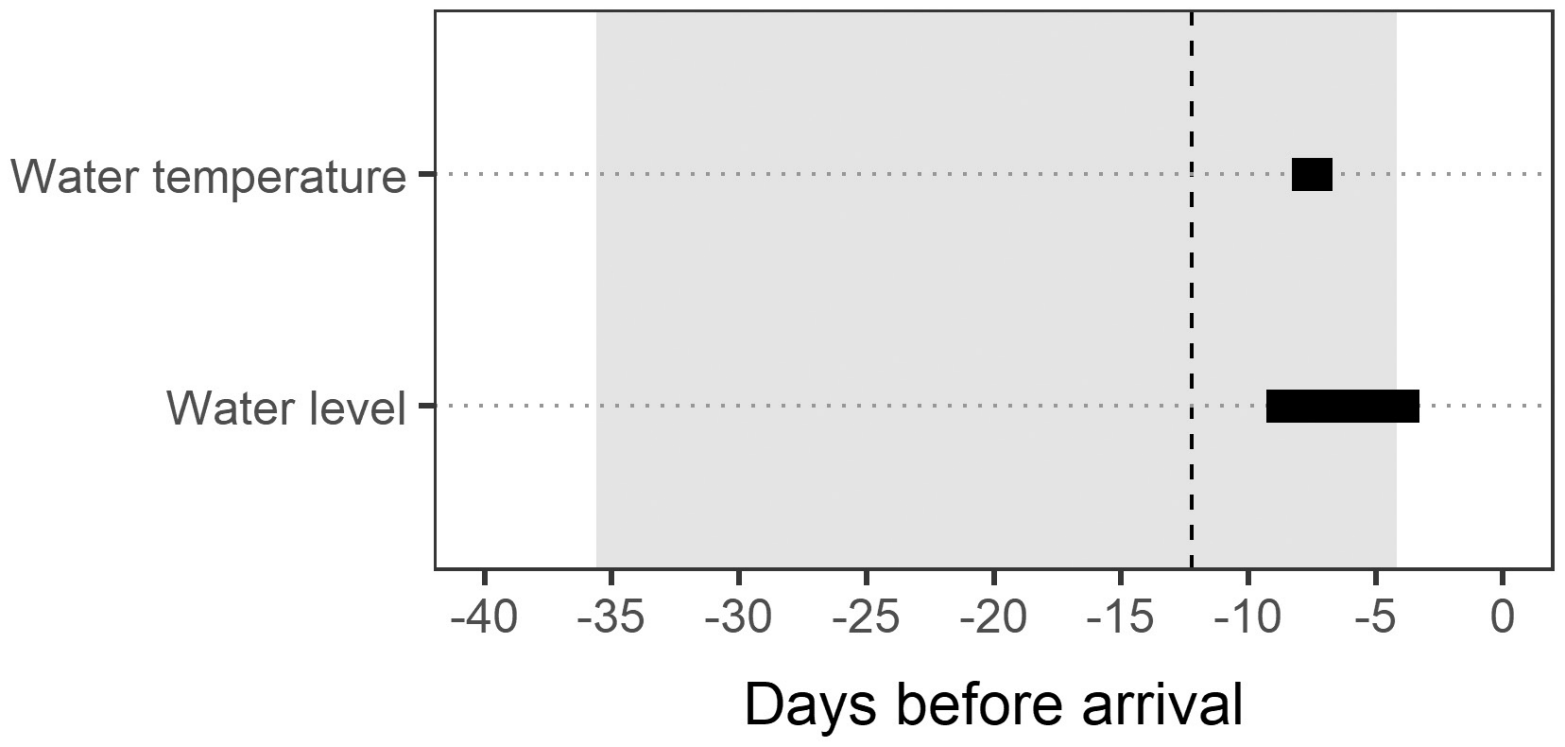
The critical time windows of water temperature and water level for female Sakhalin taimen (horizontal bars), and their average travel time from the river mouth to the spawning ground (vertical dashed line) with the 95% confidence interval (gray shading).

Mean upstream swimming speed was 1.98 km day^−1^ for females and 2.90 km day^−1^ for males, which was not significantly different between sexes. However, downstream swimming speed for females (5.68 km day^−1^) was more than double that of males (2.63 km day^−1^; *t*‐test, *p* = .046). Travel time by the upstream migrants from the river mouth to the mid‐point of tributary sites was estimated to be 12.2 days (95% CI: 4.2–35.6 days) for females (Figure 6) and 8.3 days (95% CI: 1.9–36.1 days) for males.

### 3.5 Social interactions during ascent and descent by migrants

There were 19 comigrating pairs of female Sakhalin taimen ascending through Site L during MS1, of which only two pairs entered the same tributaries for spawning. This was less than half the number (4.9) expected by chance, but the difference was not significant (*p* = .157, Table 2). Similarly, only three female pairs departed from the same tributaries, out of 22 comigrating pairs descending through Site L during MS4, which was half the expected number but also not significant (*p* = .204). The numbers of male pairs of comigrating spawners and post‐spawners entering or departing from the same tributaries, respectively, were not less than or greater than those expected by chance (*p* > .05).

**TABLE 2:** Randomization test results of the social interaction hypothesis, showing the observed numbers of comigrating pairs, and of observed and simulated (mean ± SD) pairs that entered or departed from the same tributaries.

Migration	Sex	Comigrating pairs	Observed pairs to/from same trib.	Simulated pairs to/from same trib.	*p*
Ascent (MS1)	Female	19	2	4.9 ± 1.9	.157
	Male	6	3	2.2 ± 1.1	.790
Descent (MS4)	Female	22	3	6.0 ± 2.1	.204
	Male	7	3	3.0 ± 1.3	1.000

## 4 DISCUSSION

Male Sakhalin taimen returned to the spawning grounds of the Karibetsu River several days earlier than their female counterparts. Conversely, the males departed from these sites later than the females after spawning, resulting in a longer stream residence for males. Earlier arrival at breeding sites by males (protandry) has been documented for some migratory fishes (e.g. Sinnatamby et al., 2018; Šmejkal et al., 2017; Tibblin et al., 2016), and is generally explained by the mate opportunity hypothesis that predicts that earlier arriving males of polygynous species at breeding areas increase their opportunity to mate with females (Morbey & Ydenberg, 2001). The earlier arrival and longer residence at the spawning grounds by the male Sakhalin taimen likely resulted in their lower rate of spawning across consecutive years (Fukushima & Rand, 2021). Therefore, even though the operational sex ratio can be temporarily skewed toward males earlier and again later in the season, the overall sex ratio of active spawners was female biased, according to our fish sampling and videotaped records of individual fish (Rand & Fukushima, 2014).

The number of tributaries Sakhalin taimen entered for spawning significantly influenced departure timings from tributary sites, such that individuals, especially males, spawning in two tributaries were on average later to depart from the last tributary than those spawning in a single tributary. However, subsequent arrival downstream at Site L was not influenced by the number of tributaries they entered, indicating that fish spawning in multiple tributaries tended to catch up with individuals with which they previously comigrated before spawning and restore cohesive groups during the downstream migration.

Sakhalin taimen migration timing was also different at the diel scale, between sexes and between ascent and descent in females. Male Sakhalin taimen were largely diurnal during both ascent and descent of the mainstem of the Karibetsu River, whereas females were diurnal during ascent but more likely to be nocturnal during descent. This may be explained by the propensity of male salmonids to be less risk averse than females during the spawning season (Fleming, 1996). Our model predicted that both female and male spawners and post‐spawners tended to migrate during night‐time later in the season. Decreased water levels, especially in small streams, would increase the visibility of these individuals to predators, such as bears and raptors, during daytime (Finlay et al., 2020), which is a likely explanation for our modeling results.

Female Sakhalin taimen displayed remarkable intra‐individual repeatability in seasonal migration timing between 2018 and 2019. Their departure timing was repeatable even at the diel scale during MS3. Furthermore, the females descended the mainstem in a chronological order very similar to that observed during their ascent, despite most of them spawning in different tributaries in the interim. Therefore, Sakhalin taimen, at least females, can be characterized by highly synchronized movements during the spawning migration, and strong fidelity to arrival and departure timings at specific waypoints along their migration pathways. Meta‐analysis on studies of behavioral repeatability in various animal taxa have revealed that, except for mate preference, females are generally more consistent in their behavior than males (Bell et al., 2009). Although our repeatability estimates were based only on a 2‐year dataset, they were estimated for multiple migration stages encompassing spawning and post‐spawning periods, unlike in previous studies (e.g. Brodersen et al., 2012; Tibblin et al., 2016).

Individual‐level consistency in migratory behavior has been rarely quantified for salmonids despite the long history of scientific investigation into their migration at the species or population level. This is in part due to the fact that the literature on salmonid migrations has largely focused on Pacific salmon (*Oncorhynchus* spp.), which nearly all die after the first spawning (Groot & Margolis, 1991). Even well‐studied iteroparous salmonids have rates of repeat spawning so low (e.g. <10% in steelhead *O. mykiss* (Keefer et al., 2008; Leider et al., 1986), 11% in Atlantic salmon *Salmo salar* (Fleming, 1998)) that they also are considered functionally semelparous (Penny & Moffitt, 2013), making it impractical to investigate their long‐term behavioral consistency in spawning at the individual level.

There was a significant correlation in both water temperature and water level measured in the Karibetsu mainstem between 2018 and 2019 in specific time windows relative to the arrival timing of female Sakhalin taimen in the spawning tributaries. Apart from the statistical significance of the correlations, the two critical time windows overlapped with similar time lags (8.3 and 9.3 days), indicating that individual females became responsive to these environmental signals during similar time periods. The estimated mean travel time (approx. 12 days) by the females from the river mouth to the spawning ground exceeded the time lags of the critical time windows, leading to a counterintuitive conclusion that fish made the decision to initiate upstream migration and left the river mouth “before” the environmental conditions were met. However, it should be noted that this travel time was most likely over‐estimated because it was derived by extrapolating the observed swimming speed (1.98 km day^−1^) between waypoints in the headwater area. This speed could be biased low given that fish are expected to swim slower through upper river reaches (Gauld et al., 2016) due to steeper elevation gradients, faster currents, and more natural and unnatural obstacles, like debris jams and culverts, than they encounter in the lower reaches of the Karibetsu mainstem. If a more realistic (i.e. faster) estimate of swimming speed was available for the lower reaches, their travel time would likely have been much shorter and coincided more closely with the critical time windows than what is displayed in Figure 6. Our presumption that the Sakhalin taimen spawners started upstream migration from the river mouth is justifiable according to a provenance study based on stable isotope analysis of otoliths (Fukushima et al., 2019). Strontium isotope ratios in the otolith samples of juvenile Sakhalin taimen collected throughout the Sarufutsu River drainage indicated that their mothers were inhabiting brackish waters prior to their spawning migration.

The time scale that Sakhalin taimen responded to temperature and water level appears to be different. The time window during which the fish responded to water level was much longer (6 days) than that for temperature (1.7 days), suggesting that river flow not only cued the start of migration but also continually influenced fish movements en route to the spawning ground, whereas water temperature cued migration with little influence thereafter. Simmons et al. (2021) observed that out‐migrating Atlantic salmon smolts were significantly influenced by changes in water temperature early in the run with much less influence afterwards, but that changes in river discharge exerted stronger influences on the migration toward the end of the run.

Although water temperature and stream discharge have long been recognized as the key drivers of salmonid migrations (Dahl et al., 2004; Quinn et al., 1997; Rand et al., 2006), few studies have investigated individual variability in response to these environmental drivers. To our knowledge no studies have examined how individuals might respond uniquely to water temperature, but given the influence of river flow on migration speed and successful passage, it is plausible that they respond differently among individuals to river flows that cue migration. Body size likely plays an important role, in which migration of larger individuals is cued at higher river flows. Under a scenario of a falling hydrograph, during which salmonids commonly ascend rivers for spawning (Jonsson et al., 2018; Rand et al., 2006), the migration of larger fish upriver could be cued earlier in the season. Rand and Fukushima (2014) noted a trend of decreasing size of Sakhalin taimen migrating upstream on the basis of sonar observations. However, the earlier arrival of larger fish may be simply due to the greater capability of larger fish to migrate faster (Jonsson et al., 1991).

We found no evidence that coordinated movements and repeatable behaviors of Sakhalin taimen were rooted in social interactions. Although there were comigrating, same‐sex pairs both ascending and descending the Karibetsu mainstem, we found no evidence that these pairs were cohesive during tributary entry or departure. However, the slightly smaller number of female pairs spawning in the same tributaries than expected by chance (albeit non‐significant) may be indicative of some degree of conspecific repulsion and may help minimize competition for spawning space and thus redd superimposition (Fukushima et al., 1998). It should be emphasized that although the splitting of comigrating individuals into different tributaries could be viewed as straying, between‐year consecutive return rates to the same tributaries were high (>80%) and likely a result of fine‐tuned homing capabilities (Fukushima & Rand, 2021). The relatively small sample size of tagged individuals as well as the limited spatial extent over which fish movements were examined, relative to the species' entire migration range, may have made it difficult to identify social interactions in the migratory behavior of this species.

Female and male Sakhalin taimen consistently differed in migration behaviors including seasonal and diel timings of arrival and departure, the degree to which the migration timings were coordinated and repeatable, reliance on environmental cues to initiate migration, the frequency of comigration, and swimming speed. Furthermore, female Sakhalin taimen have a higher rate of consecutive spawning than males (Fukushima & Rand, 2021). All these behavioral differences between sexes point to the hypothesis that female and male Sakhalin taimen may have different degrees of migratory connectivity (Webster et al., 2002). Although our study focused on the spawning migration period, we suspect that migratory connectivity would also be an important process during other life‐history stages. Migratory connectivity—the extent to which individuals of a migratory population behave in unison (Torniainen et al., 2014)—has been suggested to operate over the entire life cycle in some salmonid populations. For example, individual coded‐wire‐tagged steelhead released as juveniles at similar times and locations were recovered in the same fishing operation in distant locations in the North Pacific Ocean (McKinnell et al., 1997). Schools of migratory brook trout *S. fontinalis* contained significantly more kin than expected by chance for periods of up to 4 years (Fraser et al., 2005).

This migratory connectivity can be reinforced as individuals age and become more experienced (Tibblin et al., 2016), or it can be passed on to succeeding generations through learning by recruits or younger individuals from older and more experienced ones (MacCall et al., 2019). Thus, the connectivity can differ between different age‐groups and developmental or life‐history stages, a phenomenon known as differential migration (Briedis & Bauer, 2018). Differential migration between sexes in salmonids is exemplified by the migration protandry discussed above. Furthermore, a higher likelihood of adopting an anadromous life history in females than in males, a pattern often observed in salmonids with facultative migratory strategies, is an extreme example of sex‐specific differential migration (Ferguson et al., 2019; Jonsson & Jonsson, 1993). Although anadromy is not obligatory, and resident populations of Sakhalin taimen exist, depending on river systems (Zimmerman et al., 2012), facultative or partial migration is yet to be reported from any rivers with the species, including the Karibetsu River. Nonetheless, our observations in this and previous studies suggest that even though Sakhalin taimen are broadly categorized as anadromous in this river system, there seems to be sex‐specific differential migration in which females likely have stronger migratory connectivity than their male counterparts, enabling the females to better minimize variability in migration timing and form cohesive groups of migrants composed of the same members across years. The causes and consequences of the observed coordinated movements, migratory consistency, and the associated between‐sex differences in migratory behaviors in Sakhalin taimen would be better elucidated by further research focusing on migratory connectivity, not just during the breeding stage but also during non‐breeding stages of their life.

## 5 CONCLUSIONS

Spawning migration by the endangered Sakhalin taimen was characterized by coordinated movements within season and repeatable timings of arrival and departure from the spawning ground across seasons. Such behavioral consistency was more pronounced in females than males and was best explained by variations in water temperature and river flow that the females experienced in the estuarine habitat before arriving at the spawning ground. Combined with our previous findings, we demonstrated that Sakhalin taimen possess strong fidelity to both time and location of reproductive activities that enable individuals of the species, especially females, to maintain migratory connectivity within and between spawning seasons.

## AUTHOR CONTRIBUTIONS

**Michio Fukushima:** Conceptualization (equal); data curation (lead); formal analysis (lead); funding acquisition (equal); investigation (equal); methodology (equal); project administration (lead); resources (equal); visualization (lead); writing – original draft (lead); writing – review and editing (equal). **Peter S. Rand:** Conceptualization (equal); data curation (supporting); formal analysis (supporting); funding acquisition (equal); investigation (equal); methodology (equal); project administration (supporting); resources (equal); visualization (supporting); writing – original draft (supporting); writing – review and editing (equal).

## CONFLICT OF INTEREST STATEMENT

The authors have no conflicts of interest to declare.

### OPEN RESEARCH BADGES

This article has earned an Open Data badge for making publicly available the digitally‐shareable data necessary to reproduce the reported results. The data is available at [https://doi.org/10.5061/dryad.m0cfxpp7c].

## Supporting information


Figure S1

## Data Availability

All data and R code that support the findings of this study are available in Dryad at https://doi.org/10.5061/dryad.m0cfxpp7c.
