# Supplementary material for: Individual variation in spawning migration timing in a salmonid fish—Exploring roles of environmental and social cues
Source: Ecol Evol. 2023 May 17;13(5):e10101. doi: 10.1002/ece3.10101 (PMC10191801; doi:10.1002/ece3.10101)
Supplement: Supplementary file 1 — Figure S1 [file ECE3-13-e10101-s001.docx]

# Appendix


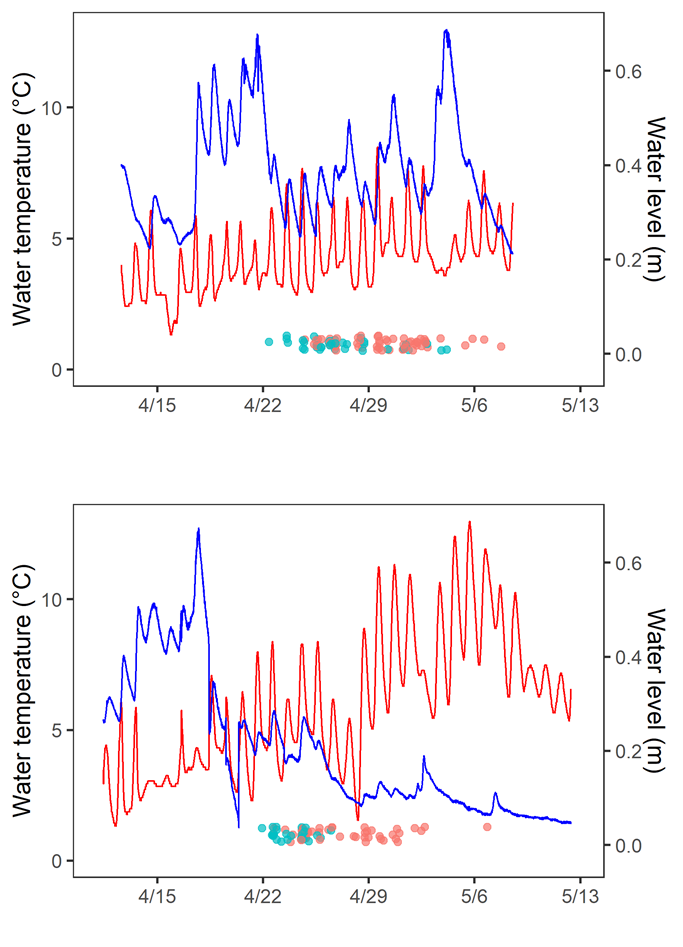


**FIGURE S1** Upstream migration timing by female (red circle) and male (blue circle) Sakhalin taimen recorded at Site L in 2018 (top) and 2019 (bottom). The circles are jittered for clarity. Water temperature (red line) and water level (blue line) are superimposed.
